# [μ-Bis(diphenyl­phosphan­yl)methane-1:2κ^2^ *P*:*P*′]nona­carbonyl-1κ^3^ *C*,2κ^3^ *C*,3κ^3^ *C*-[tris­(biphenyl-4-yl)arsane-3κ*As*]-*triangulo*-triruthenium(0)

**DOI:** 10.1107/S160053681100078X

**Published:** 2011-01-15

**Authors:** Omar bin Shawkataly, Imthyaz Ahmed Khan, Siti Syaida Sirat, Chin Sing Yeap, Hoong-Kun Fun

**Affiliations:** a Chemical Sciences Programme, School of Distance Education, Universiti Sains Malaysia, 11800 USM, Penang, Malaysia; b X-ray Crystallography Unit, School of Physics, Universiti Sains Malaysia, 11800 USM, Penang, Malaysia

## Abstract

In the title *triangulo*-triruthenium compound, [Ru_3_(C_36_H_27_As)(C_25_H_22_P_2_)(CO)_9_], the bis­(diphenyl­phosphan­yl)methane ligand bridges an Ru—Ru bond and the monodentate arsine ligand bonds to the third Ru atom. Both the arsine and phosphine ligands are equatorial with respect to the Ru_3_ triangle. In addition, each Ru atom carries one equatorial and two axial terminal carbonyl ligands. In each biphenyl unit, the phenyl rings are twisted from each other, making dihedral angles of 51.22 (18), 42.94 (16) and 26.95 (16)°. The arsine-substituted phenyl rings make dihedral angles of 61.22 (15), 87.17 (15) and 83.32 (15)° with each other. The dihedral angles between the two benzene rings are 85.52 (18) and 81.77 (15)° for the two diphenyl­phosphanyl groups, respectively. In the crystal, mol­ecules are linked into dimers by inter­molecular C—H⋯O hydrogen bonds. Weak inter­molecular C—H⋯π and π–π [centroid–centroid distance = 3.6981 (18) Å] inter­actions stabilize the crystal structure.

## Related literature

For general background to *triangulo*-triruthenium derivatives, see: Bruce *et al.* (1985 ▶, 1988*a* ▶,*b* ▶). For related structures, see: Shawkataly *et al.* (2011*a* ▶,*b* ▶). For the synthesis of Ru_3_(CO)_10_(μ-Ph_2_PCH_2_PPh_2_), see: Bruce *et al.* (1983 ▶). For the stability of the temperature controller used in the data collection, see: Cosier & Glazer (1986 ▶).
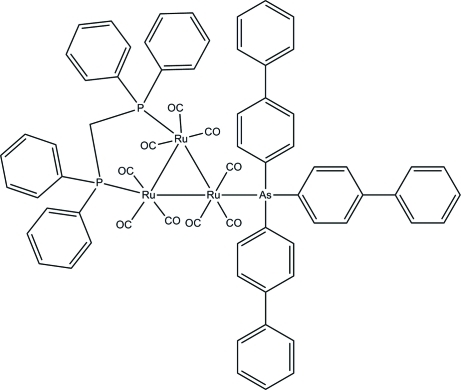


## Experimental

### 

#### Crystal data

[Ru_3_(C_36_H_27_As)(C_25_H_22_P_2_)(CO)_9_]
*M* *_r_* = 1474.16
Triclinic, 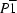

*a* = 10.8435 (7) Å
*b* = 12.6134 (8) Å
*c* = 22.4695 (15) Å
α = 81.029 (1)°
β = 82.769 (1)°
γ = 79.265 (1)°
*V* = 2967.6 (3) Å^3^
*Z* = 2
Mo *K*α radiation
μ = 1.42 mm^−1^
*T* = 100 K
0.50 × 0.17 × 0.02 mm

#### Data collection

Bruker APEXII DUO CCD area-detector diffractometer
Absorption correction: multi-scan (*SADABS*; Bruker, 2009 ▶) *T*_min_ = 0.539, *T*_max_ = 0.967
50392 measured reflections
13475 independent reflections
11159 reflections with *I* > 2σ(*I*)
*R*_int_ = 0.039

#### Refinement

*R*[*F* ^2^ > 2σ(*F* ^2^)] = 0.030
*wR*(*F* ^2^) = 0.085
*S* = 1.11
13475 reflections
766 parameters
H-atom parameters constrained
Δρ_max_ = 0.91 e Å^−3^
Δρ_min_ = −0.67 e Å^−3^

### 

Data collection: *APEX2* (Bruker, 2009 ▶); cell refinement: *SAINT* (Bruker, 2009 ▶); data reduction: *SAINT*; program(s) used to solve structure: *SHELXTL* (Sheldrick, 2008 ▶); program(s) used to refine structure: *SHELXTL*; molecular graphics: *SHELXTL*; software used to prepare material for publication: *SHELXTL* and *PLATON* (Spek, 2009 ▶).

## Supplementary Material

Crystal structure: contains datablocks global, I. DOI: 10.1107/S160053681100078X/ng5095sup1.cif

Structure factors: contains datablocks I. DOI: 10.1107/S160053681100078X/ng5095Isup2.hkl

Additional supplementary materials:  crystallographic information; 3D view; checkCIF report

## Figures and Tables

**Table 1:** Hydrogen-bond geometry (Å, °) *Cg*1 and *Cg*2 are the centroids of the C7–C12 and C26–C31 benzene rings, respectively.

*D*—H⋯*A*	*D*—H	H⋯*A*	*D*⋯*A*	*D*—H⋯*A*
C46—H46*A*⋯O9^i^	0.93	2.56	3.249 (4)	131
C21—H21*A*⋯*Cg*1	0.93	2.95	3.707 (4)	139
C24—H24*A*⋯*Cg*2^ii^	0.93	2.92	3.582 (4)	129

Symmetry codes: (i) 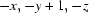; (ii) 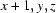.

**Fig. 1.**
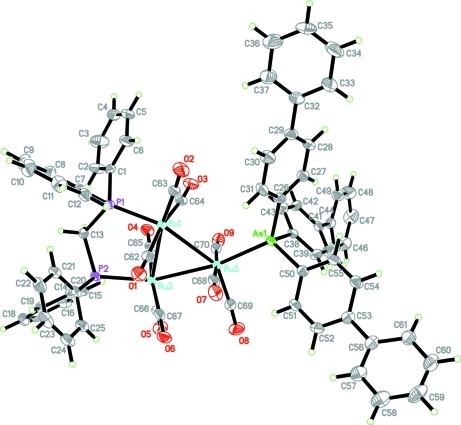
The molecular structure of the title compound with 50% probability ellipsoids for non-H atoms.

**Fig. 2.**
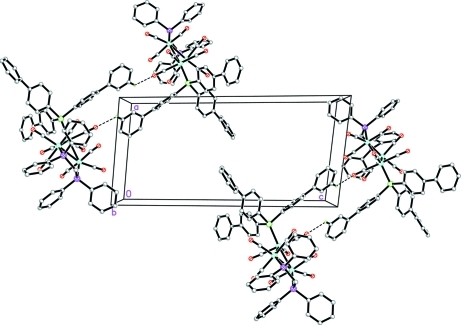
The crystal packing of the title compound, viewed down the b axis, showing the molecules are linked into dimers. Hydrogen atoms not involved in the hydrogen-bonding (dashed lines) have been omitted for clarity.

## supplementary crystallographic information

### Comment

A large number of substituted derivatives, Ru_3_(CO)_12-n_*L*_n_ (*L* = group 15 ligand) have been reported (Bruce *et al.*, 1985 1988*a*,*b*). As part of our study on the substitution of transition metal-carbonyl clusters with mixed-ligand complexes, herein we report the synthesis and structure of the title compound.

The bis(diphenylphosphanyl)methane ligand bridges the Ru1–Ru2 bond and the monodentate phosphine ligand bonds to the Ru3 atom. Both phosphine and arsine ligands are equatorial with respect to the Ru_3_ triangle. Additionally, each Ru atom carries one equatorial and two axial terminal carbonyl ligands (Fig 1). The conformation of the title compound is very identical to its closely related structure (Shawkataly *et al.*, 2011*b*). Both phenyl rings of biphenyl (C26–C31/C32–C37, C38–C43/C44–C49 and C50–C55/C56–C61) make dihedral angles of 51.22 (18), 42.94 (16) and 26.95 (16)° from each other respectively are more twisted from each other compare to the reported monodentate arsine ligand (Shawkataly *et al.*, 2011*a*). The arsine-substituted phenyl rings make dihedral angles (C26–C31/C38–C43, C26–C31/C50–C55 and C38–C43/C50–C55) of 61.22 (15), 87.17 (15) and 83.32 (15)° with each other respectively. The dihedral angles between the two benzene rings (C1–C6/C7–C12 and C14–C19/C20–C25) are 85.52 (18) and 81.77 (15)° for the two diphenylphosphanyl groups respectively.

In the crystal packing, the molecules are linked into dimers by intermolecular C46—H46A···O9 hydrogen bonds (Fig. 2, Table 1). Weak intermolecular C—H···π (Table 1) and *Cg*3···*Cg*3 interactions stabilize the crystal structure. *Cg*3···*Cg*3^iii^ = 3.6981 (18) Å; *Cg*3 is centroid of C14–C19; (iii) 2 - *x*, -*y*, -*z*.

### Experimental

All manipulations were performed under a dry oxygen-free nitrogen atmosphere using standard Schlenk techniques. All solvents were dried over sodium and distilled from sodium benzophenone ketyl under dry oxygen free nitrogen. Tri([1,1'-biphenyl]-4-yl)arsine is prepared by the reaction of AsCl_3_ with biphenyl magnesiumbromide in THF and Ru_3_(CO)_10_(µ-Ph_2_PCH_2_PPh_2_) (Bruce *et al.*, 1983) was prepared by reported procedure. The title compound was obtained by refluxing equimolar quantities of Ru_3_(CO)_10_(µ-Ph_2_PCH_2_PPh_2_) and tri([1,1'-biphenyl]-4-yl)arsine in hexane under nitrogen atmosphere. Crystals suitable for X-ray diffraction were grown by slow solvent / solvent diffusion of CH_3_OH into CH_2_Cl_2_.

### Refinement

All hydrogen atoms were positioned geometrically and refined using a riding model with C—H = 0.93 or 0.97 Å and *U*_iso_(H) = 1.2*U*_eq_(C). The maximum and minimum residual electron density peaks of 0.91 and -0.67 e Å^-3^ were located 1.45 and 0.91 Å from the O8 and Ru1 atom, respectively.

### Crystal data

**Table:**

[Ru_3_(C_36_H_27_As)(C_25_H_22_P_2_)(CO)_9_]	*Z* = 2
*M**_r_* = 1474.16	*F*(000) = 1472
Triclinic, *P*1	*D*_x_ = 1.650 Mg m^−^^3^
Hall symbol: -P 1	Mo *K*α radiation, λ = 0.71073 Å
*a* = 10.8435 (7) Å	Cell parameters from 9028 reflections
*b* = 12.6134 (8) Å	θ = 2.8–29.9°
*c* = 22.4695 (15) Å	µ = 1.42 mm^−^^1^
α = 81.029 (1)°	*T* = 100 K
β = 82.769 (1)°	Plate, brown
γ = 79.265 (1)°	0.50 × 0.17 × 0.02 mm
*V* = 2967.6 (3) Å^3^	

### Data collection

**Table:**

Bruker APEXII DUO CCD area-detector diffractometer	13475 independent reflections
Radiation source: fine-focus sealed tube	11159 reflections with *I* > 2σ(*I*)
graphite	*R*_int_ = 0.039
φ and ω scans	θ_max_ = 27.5°, θ_min_ = 1.8°
Absorption correction: multi-scan (*SADABS*; Bruker, 2009)	*h* = −13→14
*T*_min_ = 0.539, *T*_max_ = 0.967	*k* = −16→16
50392 measured reflections	*l* = −29→29

### Refinement

**Table:**

Refinement on *F*^2^	Primary atom site location: structure-invariant direct methods
Least-squares matrix: full	Secondary atom site location: difference Fourier map
*R*[*F*^2^ > 2σ(*F*^2^)] = 0.030	Hydrogen site location: inferred from neighbouring sites
*wR*(*F*^2^) = 0.085	H-atom parameters constrained
*S* = 1.11	*w* = 1/[σ^2^(*F*_o_^2^) + (0.0394*P*)^2^ + 2.0751*P*] where *P* = (*F*_o_^2^ + 2*F*_c_^2^)/3
13475 reflections	(Δ/σ)_max_ = 0.001
766 parameters	Δρ_max_ = 0.91 e Å^−^^3^
0 restraints	Δρ_min_ = −0.67 e Å^−^^3^

### Special details

**Table:**

Experimental. The crystal was placed in the cold stream of an Oxford Cryosystems Cobra open-flow nitrogen cryostat (Cosier & Glazer, 1986) operating at 100.0 (1) K.
Geometry. All e.s.d.'s (except the e.s.d. in the dihedral angle between two l.s. planes) are estimated using the full covariance matrix. The cell e.s.d.'s are taken into account individually in the estimation of e.s.d.'s in distances, angles and torsion angles; correlations between e.s.d.'s in cell parameters are only used when they are defined by crystal symmetry. An approximate (isotropic) treatment of cell e.s.d.'s is used for estimating e.s.d.'s involving l.s. planes.
Refinement. Refinement of *F*^2^ against ALL reflections. The weighted *R*-factor *wR* and goodness of fit *S* are based on *F*^2^, conventional *R*-factors *R* are based on *F*, with *F* set to zero for negative *F*^2^. The threshold expression of *F*^2^ > σ(*F*^2^) is used only for calculating *R*-factors(gt) *etc*. and is not relevant to the choice of reflections for refinement. *R*-factors based on *F*^2^ are statistically about twice as large as those based on *F*, and *R*- factors based on ALL data will be even larger.

### Fractional atomic coordinates and isotropic or equivalent isotropic displacement parameters (Å^2^)

**Table:**

	*x*	*y*	*z*	*U*_iso_*/*U*_eq_	
Ru1	0.44891 (2)	−0.078490 (17)	0.251421 (10)	0.01666 (6)	
Ru2	0.59682 (2)	0.043132 (17)	0.162673 (10)	0.01608 (6)	
Ru3	0.38778 (2)	0.156754 (18)	0.228718 (10)	0.01686 (6)	
As1	0.18353 (3)	0.23145 (2)	0.280389 (13)	0.01803 (7)	
P1	0.53340 (7)	−0.23935 (6)	0.21184 (3)	0.01721 (15)	
P2	0.74739 (7)	−0.11370 (6)	0.15430 (3)	0.01714 (15)	
O1	0.6635 (2)	−0.08213 (19)	0.32987 (10)	0.0307 (5)	
O2	0.2964 (2)	−0.1792 (2)	0.36158 (10)	0.0366 (6)	
O3	0.2224 (2)	−0.04572 (19)	0.17687 (10)	0.0306 (5)	
O4	0.4449 (2)	−0.02871 (18)	0.07405 (10)	0.0278 (5)	
O5	0.6747 (3)	0.21237 (19)	0.06021 (10)	0.0377 (6)	
O6	0.7332 (2)	0.1412 (2)	0.24679 (10)	0.0315 (5)	
O7	0.5071 (2)	0.1390 (2)	0.34769 (10)	0.0325 (5)	
O8	0.4815 (3)	0.36584 (19)	0.17562 (11)	0.0404 (6)	
O9	0.2679 (2)	0.17734 (18)	0.11014 (10)	0.0300 (5)	
C1	0.4231 (3)	−0.2927 (2)	0.17412 (13)	0.0214 (6)	
C2	0.4389 (4)	−0.3083 (3)	0.11416 (15)	0.0347 (8)	
H2A	0.5104	−0.2910	0.0899	0.042*	
C3	0.3489 (4)	−0.3499 (3)	0.08924 (17)	0.0437 (10)	
H3A	0.3605	−0.3593	0.0485	0.052*	
C4	0.2444 (3)	−0.3767 (3)	0.12452 (17)	0.0345 (8)	
H4A	0.1864	−0.4072	0.1084	0.041*	
C5	0.2255 (4)	−0.3585 (3)	0.18381 (18)	0.0373 (9)	
H5A	0.1529	−0.3746	0.2075	0.045*	
C6	0.3125 (3)	−0.3169 (3)	0.20871 (16)	0.0329 (8)	
H6A	0.2978	−0.3046	0.2489	0.040*	
C7	0.5935 (3)	−0.3629 (2)	0.26236 (13)	0.0216 (6)	
C8	0.6277 (3)	−0.4617 (2)	0.23914 (14)	0.0275 (7)	
H8A	0.6143	−0.4654	0.1995	0.033*	
C9	0.6816 (4)	−0.5544 (3)	0.27470 (16)	0.0383 (9)	
H9A	0.7049	−0.6199	0.2588	0.046*	
C10	0.7004 (4)	−0.5497 (3)	0.33351 (16)	0.0392 (9)	
H10A	0.7373	−0.6118	0.3573	0.047*	
C11	0.6646 (4)	−0.4525 (3)	0.35749 (15)	0.0346 (8)	
H11A	0.6762	−0.4501	0.3975	0.041*	
C12	0.6117 (3)	−0.3592 (3)	0.32242 (14)	0.0259 (7)	
H12A	0.5883	−0.2942	0.3387	0.031*	
C13	0.6680 (3)	−0.2300 (2)	0.15242 (12)	0.0190 (6)	
H13A	0.7289	−0.2968	0.1575	0.023*	
H13B	0.6376	−0.2236	0.1130	0.023*	
C14	0.8514 (3)	−0.1181 (2)	0.08339 (13)	0.0201 (6)	
C15	0.8004 (3)	−0.0690 (2)	0.02949 (13)	0.0237 (6)	
H15A	0.7168	−0.0342	0.0304	0.028*	
C16	0.8753 (3)	−0.0727 (3)	−0.02557 (13)	0.0280 (7)	
H16A	0.8415	−0.0407	−0.0614	0.034*	
C17	0.9990 (3)	−0.1235 (2)	−0.02698 (14)	0.0278 (7)	
H17A	1.0490	−0.1246	−0.0638	0.033*	
C18	1.0501 (3)	−0.1732 (3)	0.02629 (15)	0.0296 (7)	
H18A	1.1336	−0.2083	0.0251	0.036*	
C19	0.9754 (3)	−0.1701 (2)	0.08146 (14)	0.0249 (7)	
H19A	1.0093	−0.2032	0.1171	0.030*	
C20	0.8549 (3)	−0.1593 (2)	0.21285 (13)	0.0203 (6)	
C21	0.8689 (3)	−0.2634 (3)	0.24509 (14)	0.0249 (6)	
H21A	0.8234	−0.3136	0.2361	0.030*	
C22	0.9496 (3)	−0.2942 (3)	0.29044 (15)	0.0312 (7)	
H22A	0.9573	−0.3640	0.3119	0.037*	
C23	1.0183 (3)	−0.2204 (3)	0.30354 (15)	0.0332 (8)	
H23A	1.0722	−0.2408	0.3340	0.040*	
C24	1.0076 (3)	−0.1164 (3)	0.27177 (14)	0.0302 (7)	
H24A	1.0543	−0.0671	0.2807	0.036*	
C25	0.9266 (3)	−0.0862 (3)	0.22646 (14)	0.0269 (7)	
H25A	0.9199	−0.0165	0.2049	0.032*	
C26	0.0712 (3)	0.1451 (2)	0.33399 (13)	0.0214 (6)	
C27	−0.0586 (3)	0.1666 (2)	0.33179 (13)	0.0240 (6)	
H27A	−0.0941	0.2243	0.3047	0.029*	
C28	−0.1362 (3)	0.1027 (3)	0.36984 (13)	0.0253 (7)	
H28A	−0.2231	0.1185	0.3681	0.030*	
C29	−0.0847 (3)	0.0155 (3)	0.41026 (13)	0.0260 (7)	
C30	0.0441 (3)	−0.0045 (3)	0.41272 (14)	0.0303 (7)	
H30A	0.0796	−0.0618	0.4400	0.036*	
C31	0.1223 (3)	0.0596 (3)	0.37503 (14)	0.0277 (7)	
H31A	0.2090	0.0448	0.3775	0.033*	
C32	−0.1695 (3)	−0.0550 (3)	0.44874 (14)	0.0280 (7)	
C33	−0.2758 (4)	−0.0132 (3)	0.48258 (18)	0.0440 (10)	
H33A	−0.2972	0.0619	0.4820	0.053*	
C34	−0.3534 (4)	−0.0810 (4)	0.51826 (19)	0.0497 (11)	
H34A	−0.4251	−0.0504	0.5410	0.060*	
C35	−0.3249 (4)	−0.1900 (3)	0.51981 (17)	0.0426 (10)	
H35A	−0.3751	−0.2351	0.5443	0.051*	
C36	−0.2208 (5)	−0.2332 (3)	0.48460 (19)	0.0520 (11)	
H36A	−0.2026	−0.3080	0.4837	0.062*	
C37	−0.1421 (4)	−0.1665 (3)	0.45017 (17)	0.0418 (9)	
H37A	−0.0700	−0.1975	0.4278	0.050*	
C38	0.0689 (3)	0.3128 (2)	0.22322 (13)	0.0189 (6)	
C39	0.0500 (3)	0.4262 (2)	0.21320 (14)	0.0253 (7)	
H39A	0.0863	0.4639	0.2369	0.030*	
C40	−0.0220 (3)	0.4828 (2)	0.16844 (14)	0.0255 (7)	
H40A	−0.0321	0.5585	0.1618	0.031*	
C41	−0.0798 (3)	0.4293 (2)	0.13306 (13)	0.0201 (6)	
C42	−0.0631 (3)	0.3152 (2)	0.14432 (14)	0.0240 (6)	
H42A	−0.1029	0.2777	0.1220	0.029*	
C43	0.0116 (3)	0.2581 (2)	0.18805 (14)	0.0238 (6)	
H43A	0.0238	0.1824	0.1941	0.029*	
C44	−0.1574 (3)	0.4898 (2)	0.08500 (13)	0.0229 (6)	
C45	−0.1181 (3)	0.5773 (3)	0.04611 (14)	0.0296 (7)	
H45A	−0.0429	0.5989	0.0508	0.035*	
C46	−0.1900 (4)	0.6326 (3)	0.00051 (16)	0.0387 (9)	
H46A	−0.1632	0.6909	−0.0252	0.046*	
C47	−0.3019 (4)	0.6006 (3)	−0.00657 (17)	0.0425 (9)	
H47A	−0.3497	0.6367	−0.0376	0.051*	
C48	−0.3424 (4)	0.5159 (3)	0.03199 (18)	0.0432 (9)	
H48A	−0.4188	0.4959	0.0276	0.052*	
C49	−0.2709 (3)	0.4600 (3)	0.07737 (15)	0.0311 (7)	
H49A	−0.2990	0.4021	0.1030	0.037*	
C50	0.1957 (3)	0.3424 (2)	0.32999 (13)	0.0206 (6)	
C51	0.3039 (3)	0.3891 (3)	0.32329 (14)	0.0270 (7)	
H51A	0.3696	0.3683	0.2944	0.032*	
C52	0.3153 (3)	0.4654 (3)	0.35879 (14)	0.0283 (7)	
H52A	0.3883	0.4960	0.3530	0.034*	
C53	0.2209 (3)	0.4978 (2)	0.40296 (13)	0.0244 (7)	
C54	0.1089 (3)	0.4544 (3)	0.40785 (14)	0.0293 (7)	
H54A	0.0419	0.4775	0.4356	0.035*	
C55	0.0971 (3)	0.3781 (3)	0.37216 (14)	0.0275 (7)	
H55A	0.0225	0.3501	0.3763	0.033*	
C56	0.2393 (3)	0.5731 (3)	0.44450 (13)	0.0276 (7)	
C57	0.3587 (4)	0.5795 (3)	0.45740 (15)	0.0357 (8)	
H57A	0.4278	0.5355	0.4398	0.043*	
C58	0.3789 (4)	0.6495 (3)	0.49567 (16)	0.0417 (9)	
H58A	0.4604	0.6527	0.5032	0.050*	
C59	0.2778 (5)	0.7135 (3)	0.52219 (15)	0.0448 (11)	
H59A	0.2904	0.7598	0.5485	0.054*	
C60	0.1584 (4)	0.7098 (3)	0.51025 (15)	0.0400 (10)	
H60A	0.0903	0.7540	0.5284	0.048*	
C61	0.1373 (4)	0.6409 (3)	0.47137 (15)	0.0353 (8)	
H61A	0.0556	0.6398	0.4632	0.042*	
C62	0.5866 (3)	−0.0778 (2)	0.29897 (13)	0.0233 (6)	
C63	0.3517 (3)	−0.1386 (2)	0.31993 (13)	0.0234 (6)	
C64	0.3081 (3)	−0.0515 (2)	0.20328 (13)	0.0219 (6)	
C65	0.4964 (3)	−0.0032 (2)	0.10934 (13)	0.0213 (6)	
C66	0.6478 (3)	0.1485 (2)	0.09948 (13)	0.0235 (6)	
C67	0.6797 (3)	0.1011 (2)	0.21860 (13)	0.0235 (6)	
C68	0.4649 (3)	0.1379 (2)	0.30345 (14)	0.0248 (7)	
C69	0.4426 (3)	0.2888 (2)	0.19714 (14)	0.0264 (7)	
C70	0.3137 (3)	0.1633 (2)	0.15409 (14)	0.0232 (6)	

### Atomic displacement parameters (Å^2^)

**Table:**

	*U*^11^	*U*^22^	*U*^33^	*U*^12^	*U*^13^	*U*^23^
Ru1	0.01510 (13)	0.01704 (11)	0.01680 (11)	−0.00413 (9)	0.00168 (9)	−0.00005 (8)
Ru2	0.01274 (12)	0.01755 (11)	0.01752 (11)	−0.00462 (9)	0.00147 (9)	−0.00097 (8)
Ru3	0.01466 (12)	0.01714 (11)	0.01768 (11)	−0.00304 (9)	0.00072 (9)	−0.00051 (8)
As1	0.01521 (16)	0.01817 (14)	0.02020 (14)	−0.00388 (11)	−0.00016 (11)	−0.00104 (11)
P1	0.0151 (4)	0.0171 (3)	0.0187 (3)	−0.0043 (3)	0.0000 (3)	0.0001 (3)
P2	0.0133 (4)	0.0192 (3)	0.0186 (3)	−0.0041 (3)	0.0008 (3)	−0.0017 (3)
O1	0.0280 (14)	0.0378 (13)	0.0279 (11)	−0.0057 (11)	−0.0070 (10)	−0.0052 (10)
O2	0.0341 (15)	0.0388 (13)	0.0309 (12)	−0.0096 (12)	0.0090 (11)	0.0085 (10)
O3	0.0235 (13)	0.0353 (12)	0.0332 (12)	−0.0119 (10)	−0.0074 (10)	0.0071 (10)
O4	0.0234 (13)	0.0366 (12)	0.0258 (11)	−0.0082 (10)	−0.0042 (9)	−0.0064 (9)
O5	0.0469 (17)	0.0292 (12)	0.0332 (12)	−0.0144 (12)	0.0128 (12)	0.0034 (10)
O6	0.0218 (13)	0.0428 (13)	0.0350 (12)	−0.0111 (11)	−0.0034 (10)	−0.0141 (10)
O7	0.0280 (14)	0.0446 (14)	0.0262 (12)	−0.0020 (11)	−0.0055 (10)	−0.0117 (10)
O8	0.0486 (18)	0.0282 (12)	0.0440 (14)	−0.0193 (12)	0.0108 (13)	−0.0008 (10)
O9	0.0346 (15)	0.0286 (11)	0.0248 (11)	0.0020 (10)	−0.0102 (10)	−0.0004 (9)
C1	0.0194 (16)	0.0156 (13)	0.0292 (15)	−0.0046 (12)	−0.0030 (12)	−0.0010 (11)
C2	0.034 (2)	0.046 (2)	0.0302 (17)	−0.0238 (17)	−0.0013 (15)	−0.0046 (15)
C3	0.047 (3)	0.059 (2)	0.0349 (19)	−0.026 (2)	−0.0120 (18)	−0.0083 (17)
C4	0.0232 (19)	0.0317 (17)	0.053 (2)	−0.0069 (15)	−0.0106 (16)	−0.0100 (15)
C5	0.0218 (19)	0.0364 (19)	0.058 (2)	−0.0152 (15)	0.0048 (17)	−0.0136 (17)
C6	0.027 (2)	0.0382 (18)	0.0358 (18)	−0.0144 (15)	0.0078 (15)	−0.0107 (14)
C7	0.0156 (16)	0.0218 (14)	0.0248 (14)	−0.0038 (12)	0.0014 (12)	0.0026 (11)
C8	0.0291 (19)	0.0225 (15)	0.0275 (16)	−0.0002 (13)	0.0019 (14)	−0.0015 (12)
C9	0.043 (2)	0.0271 (17)	0.0387 (19)	0.0015 (16)	0.0015 (17)	0.0010 (14)
C10	0.040 (2)	0.0328 (18)	0.0362 (19)	0.0029 (16)	−0.0016 (17)	0.0102 (15)
C11	0.032 (2)	0.0418 (19)	0.0245 (16)	−0.0019 (16)	−0.0019 (14)	0.0080 (14)
C12	0.0215 (17)	0.0275 (15)	0.0261 (15)	−0.0032 (13)	0.0018 (13)	0.0002 (12)
C13	0.0179 (16)	0.0193 (13)	0.0200 (13)	−0.0034 (12)	0.0015 (11)	−0.0053 (11)
C14	0.0195 (16)	0.0205 (13)	0.0208 (13)	−0.0077 (12)	0.0029 (12)	−0.0032 (11)
C15	0.0205 (17)	0.0269 (15)	0.0257 (15)	−0.0090 (13)	0.0022 (12)	−0.0074 (12)
C16	0.035 (2)	0.0340 (17)	0.0186 (14)	−0.0147 (15)	0.0006 (13)	−0.0056 (12)
C17	0.034 (2)	0.0262 (15)	0.0253 (15)	−0.0123 (14)	0.0090 (14)	−0.0093 (12)
C18	0.0216 (18)	0.0298 (16)	0.0351 (17)	−0.0044 (14)	0.0098 (14)	−0.0077 (13)
C19	0.0241 (18)	0.0241 (15)	0.0241 (15)	−0.0042 (13)	0.0046 (13)	−0.0019 (12)
C20	0.0133 (15)	0.0260 (14)	0.0206 (14)	−0.0006 (12)	−0.0002 (11)	−0.0047 (11)
C21	0.0176 (16)	0.0293 (16)	0.0278 (15)	−0.0048 (13)	−0.0004 (13)	−0.0041 (12)
C22	0.0228 (18)	0.0362 (18)	0.0294 (16)	0.0014 (14)	−0.0025 (14)	0.0041 (14)
C23	0.0199 (18)	0.052 (2)	0.0256 (16)	0.0000 (16)	−0.0037 (13)	−0.0055 (15)
C24	0.0190 (18)	0.0442 (19)	0.0297 (16)	−0.0073 (15)	0.0018 (13)	−0.0137 (14)
C25	0.0192 (17)	0.0348 (17)	0.0272 (15)	−0.0067 (14)	0.0010 (13)	−0.0060 (13)
C26	0.0227 (17)	0.0219 (14)	0.0195 (13)	−0.0066 (12)	0.0025 (12)	−0.0026 (11)
C27	0.0219 (17)	0.0239 (14)	0.0254 (15)	−0.0041 (13)	−0.0028 (13)	−0.0007 (12)
C28	0.0177 (17)	0.0309 (16)	0.0281 (15)	−0.0075 (13)	−0.0003 (13)	−0.0038 (13)
C29	0.0270 (18)	0.0278 (15)	0.0238 (15)	−0.0113 (14)	0.0040 (13)	−0.0029 (12)
C30	0.0278 (19)	0.0318 (17)	0.0285 (16)	−0.0064 (15)	−0.0037 (14)	0.0066 (13)
C31	0.0167 (17)	0.0321 (16)	0.0310 (16)	−0.0017 (13)	−0.0007 (13)	0.0013 (13)
C32	0.0276 (19)	0.0350 (17)	0.0219 (15)	−0.0112 (15)	−0.0025 (13)	0.0015 (13)
C33	0.037 (2)	0.039 (2)	0.052 (2)	−0.0109 (18)	0.0119 (19)	−0.0010 (17)
C34	0.033 (2)	0.058 (3)	0.052 (2)	−0.013 (2)	0.0123 (19)	0.005 (2)
C35	0.042 (2)	0.048 (2)	0.039 (2)	−0.0259 (19)	−0.0019 (18)	0.0117 (17)
C36	0.062 (3)	0.043 (2)	0.049 (2)	−0.022 (2)	0.008 (2)	0.0050 (18)
C37	0.047 (3)	0.0365 (19)	0.0382 (19)	−0.0132 (18)	0.0086 (18)	0.0015 (15)
C38	0.0135 (15)	0.0200 (13)	0.0221 (14)	−0.0033 (11)	−0.0006 (11)	0.0001 (11)
C39	0.0277 (18)	0.0208 (14)	0.0304 (16)	−0.0078 (13)	−0.0071 (14)	−0.0044 (12)
C40	0.0280 (19)	0.0185 (14)	0.0305 (16)	−0.0030 (13)	−0.0086 (14)	−0.0012 (12)
C41	0.0152 (15)	0.0216 (14)	0.0217 (14)	−0.0018 (12)	0.0017 (11)	−0.0021 (11)
C42	0.0252 (18)	0.0201 (14)	0.0282 (15)	−0.0050 (13)	−0.0053 (13)	−0.0050 (12)
C43	0.0266 (18)	0.0139 (13)	0.0301 (16)	−0.0012 (12)	−0.0037 (13)	−0.0024 (11)
C44	0.0233 (17)	0.0208 (14)	0.0240 (14)	−0.0021 (12)	−0.0015 (12)	−0.0041 (11)
C45	0.031 (2)	0.0273 (16)	0.0302 (16)	−0.0067 (14)	−0.0062 (14)	−0.0001 (13)
C46	0.047 (3)	0.0303 (17)	0.0357 (19)	−0.0045 (17)	−0.0087 (17)	0.0049 (14)
C47	0.050 (3)	0.039 (2)	0.039 (2)	−0.0014 (18)	−0.0252 (19)	0.0024 (16)
C48	0.033 (2)	0.045 (2)	0.055 (2)	−0.0071 (18)	−0.0221 (19)	−0.0046 (18)
C49	0.0277 (19)	0.0277 (16)	0.0384 (18)	−0.0057 (14)	−0.0072 (15)	−0.0021 (14)
C50	0.0185 (16)	0.0218 (14)	0.0205 (13)	−0.0026 (12)	−0.0019 (12)	−0.0011 (11)
C51	0.0190 (17)	0.0313 (16)	0.0311 (16)	−0.0063 (13)	0.0048 (13)	−0.0090 (13)
C52	0.0239 (18)	0.0298 (16)	0.0333 (17)	−0.0120 (14)	0.0016 (14)	−0.0055 (13)
C53	0.0293 (19)	0.0239 (14)	0.0199 (14)	−0.0069 (13)	−0.0014 (13)	−0.0008 (11)
C54	0.0250 (19)	0.0377 (18)	0.0259 (15)	−0.0096 (15)	0.0064 (13)	−0.0089 (13)
C55	0.0208 (18)	0.0334 (17)	0.0293 (16)	−0.0099 (14)	0.0028 (13)	−0.0050 (13)
C56	0.036 (2)	0.0253 (15)	0.0207 (14)	−0.0082 (14)	0.0017 (13)	−0.0024 (12)
C57	0.042 (2)	0.0356 (18)	0.0314 (17)	−0.0098 (17)	−0.0068 (16)	−0.0040 (14)
C58	0.058 (3)	0.039 (2)	0.0324 (18)	−0.0136 (19)	−0.0132 (18)	−0.0045 (15)
C59	0.081 (3)	0.0333 (19)	0.0253 (17)	−0.022 (2)	−0.0015 (19)	−0.0067 (14)
C60	0.058 (3)	0.0360 (19)	0.0258 (17)	−0.0155 (19)	0.0133 (17)	−0.0085 (14)
C61	0.040 (2)	0.0327 (17)	0.0325 (17)	−0.0123 (16)	0.0098 (16)	−0.0065 (14)
C62	0.0246 (18)	0.0205 (14)	0.0233 (14)	−0.0050 (13)	0.0027 (13)	−0.0017 (11)
C63	0.0204 (17)	0.0232 (14)	0.0258 (15)	−0.0040 (13)	−0.0025 (13)	−0.0007 (12)
C64	0.0205 (17)	0.0211 (14)	0.0224 (14)	−0.0076 (12)	0.0029 (12)	0.0034 (11)
C65	0.0173 (16)	0.0223 (14)	0.0214 (14)	−0.0031 (12)	0.0046 (12)	0.0004 (11)
C66	0.0183 (16)	0.0256 (15)	0.0266 (15)	−0.0067 (13)	0.0043 (12)	−0.0058 (12)
C67	0.0162 (16)	0.0255 (15)	0.0262 (15)	−0.0022 (12)	0.0049 (12)	−0.0032 (12)
C68	0.0181 (17)	0.0275 (15)	0.0271 (16)	−0.0030 (13)	0.0030 (13)	−0.0034 (12)
C69	0.0234 (18)	0.0246 (15)	0.0302 (16)	−0.0040 (13)	0.0029 (13)	−0.0049 (12)
C70	0.0225 (17)	0.0181 (13)	0.0270 (15)	−0.0023 (12)	0.0012 (13)	−0.0011 (11)

### Geometric parameters (Å, °)

**Table:**

Ru1—C63	1.892 (3)	C22—H22A	0.9300
Ru1—C64	1.930 (3)	C23—C24	1.385 (5)
Ru1—C62	1.944 (3)	C23—H23A	0.9300
Ru1—P1	2.3265 (8)	C24—C25	1.390 (5)
Ru1—Ru2	2.8389 (3)	C24—H24A	0.9300
Ru1—Ru3	2.8956 (4)	C25—H25A	0.9300
Ru2—C66	1.891 (3)	C26—C31	1.384 (4)
Ru2—C67	1.933 (3)	C26—C27	1.388 (5)
Ru2—C65	1.935 (3)	C27—C28	1.395 (4)
Ru2—P2	2.3345 (8)	C27—H27A	0.9300
Ru2—Ru3	2.8285 (3)	C28—C29	1.390 (4)
Ru3—C69	1.878 (3)	C28—H28A	0.9300
Ru3—C68	1.931 (3)	C29—C30	1.379 (5)
Ru3—C70	1.932 (3)	C29—C32	1.499 (4)
Ru3—As1	2.4612 (4)	C30—C31	1.397 (4)
As1—C38	1.940 (3)	C30—H30A	0.9300
As1—C50	1.952 (3)	C31—H31A	0.9300
As1—C26	1.959 (3)	C32—C33	1.365 (5)
P1—C1	1.826 (3)	C32—C37	1.378 (5)
P1—C7	1.843 (3)	C33—C34	1.402 (5)
P1—C13	1.858 (3)	C33—H33A	0.9300
P2—C20	1.823 (3)	C34—C35	1.348 (6)
P2—C14	1.835 (3)	C34—H34A	0.9300
P2—C13	1.843 (3)	C35—C36	1.370 (6)
O1—C62	1.139 (4)	C35—H35A	0.9300
O2—C63	1.150 (4)	C36—C37	1.391 (5)
O3—C64	1.149 (4)	C36—H36A	0.9300
O4—C65	1.142 (4)	C37—H37A	0.9300
O5—C66	1.146 (4)	C38—C43	1.393 (4)
O6—C67	1.138 (4)	C38—C39	1.393 (4)
O7—C68	1.149 (4)	C39—C40	1.378 (4)
O8—C69	1.145 (4)	C39—H39A	0.9300
O9—C70	1.136 (4)	C40—C41	1.391 (4)
C1—C2	1.377 (4)	C40—H40A	0.9300
C1—C6	1.401 (4)	C41—C42	1.403 (4)
C2—C3	1.400 (5)	C41—C44	1.479 (4)
C2—H2A	0.9300	C42—C43	1.380 (4)
C3—C4	1.364 (5)	C42—H42A	0.9300
C3—H3A	0.9300	C43—H43A	0.9300
C4—C5	1.371 (5)	C44—C49	1.390 (5)
C4—H4A	0.9300	C44—C45	1.394 (4)
C5—C6	1.375 (5)	C45—C46	1.385 (5)
C5—H5A	0.9300	C45—H45A	0.9300
C6—H6A	0.9300	C46—C47	1.381 (6)
C7—C8	1.395 (4)	C46—H46A	0.9300
C7—C12	1.397 (4)	C47—C48	1.370 (5)
C8—C9	1.385 (5)	C47—H47A	0.9300
C8—H8A	0.9300	C48—C49	1.382 (5)
C9—C10	1.374 (5)	C48—H48A	0.9300
C9—H9A	0.9300	C49—H49A	0.9300
C10—C11	1.385 (5)	C50—C51	1.389 (4)
C10—H10A	0.9300	C50—C55	1.394 (4)
C11—C12	1.381 (4)	C51—C52	1.374 (4)
C11—H11A	0.9300	C51—H51A	0.9300
C12—H12A	0.9300	C52—C53	1.384 (4)
C13—H13A	0.9700	C52—H52A	0.9300
C13—H13B	0.9700	C53—C54	1.408 (4)
C14—C19	1.380 (4)	C53—C56	1.485 (4)
C14—C15	1.401 (4)	C54—C55	1.377 (4)
C15—C16	1.394 (4)	C54—H54A	0.9300
C15—H15A	0.9300	C55—H55A	0.9300
C16—C17	1.373 (5)	C56—C57	1.381 (5)
C16—H16A	0.9300	C56—C61	1.399 (5)
C17—C18	1.391 (5)	C57—C58	1.386 (5)
C17—H17A	0.9300	C57—H57A	0.9300
C18—C19	1.394 (4)	C58—C59	1.365 (6)
C18—H18A	0.9300	C58—H58A	0.9300
C19—H19A	0.9300	C59—C60	1.365 (6)
C20—C21	1.389 (4)	C59—H59A	0.9300
C20—C25	1.400 (4)	C60—C61	1.390 (5)
C21—C22	1.389 (5)	C60—H60A	0.9300
C21—H21A	0.9300	C61—H61A	0.9300
C22—C23	1.380 (5)		
			
C63—Ru1—C64	91.69 (13)	C23—C22—C21	119.6 (3)
C63—Ru1—C62	91.95 (13)	C23—C22—H22A	120.2
C64—Ru1—C62	169.84 (12)	C21—C22—H22A	120.2
C63—Ru1—P1	98.00 (9)	C22—C23—C24	120.6 (3)
C64—Ru1—P1	91.94 (9)	C22—C23—H23A	119.7
C62—Ru1—P1	96.94 (9)	C24—C23—H23A	119.7
C63—Ru1—Ru2	168.92 (9)	C23—C24—C25	119.5 (3)
C64—Ru1—Ru2	93.18 (8)	C23—C24—H24A	120.3
C62—Ru1—Ru2	81.66 (8)	C25—C24—H24A	120.3
P1—Ru1—Ru2	91.782 (19)	C24—C25—C20	120.9 (3)
C63—Ru1—Ru3	112.71 (9)	C24—C25—H25A	119.5
C64—Ru1—Ru3	76.03 (8)	C20—C25—H25A	119.5
C62—Ru1—Ru3	93.82 (9)	C31—C26—C27	118.9 (3)
P1—Ru1—Ru3	147.04 (2)	C31—C26—As1	119.3 (2)
Ru2—Ru1—Ru3	59.098 (8)	C27—C26—As1	121.9 (2)
C66—Ru2—C67	89.89 (13)	C26—C27—C28	120.7 (3)
C66—Ru2—C65	90.22 (12)	C26—C27—H27A	119.7
C67—Ru2—C65	173.34 (13)	C28—C27—H27A	119.7
C66—Ru2—P2	105.29 (10)	C29—C28—C27	120.5 (3)
C67—Ru2—P2	96.30 (10)	C29—C28—H28A	119.8
C65—Ru2—P2	90.10 (9)	C27—C28—H28A	119.8
C66—Ru2—Ru3	104.86 (10)	C30—C29—C28	118.5 (3)
C67—Ru2—Ru3	78.76 (9)	C30—C29—C32	122.0 (3)
C65—Ru2—Ru3	94.77 (9)	C28—C29—C32	119.5 (3)
P2—Ru2—Ru3	149.428 (19)	C29—C30—C31	121.4 (3)
C66—Ru2—Ru1	163.01 (10)	C29—C30—H30A	119.3
C67—Ru2—Ru1	96.62 (8)	C31—C30—H30A	119.3
C65—Ru2—Ru1	81.51 (8)	C26—C31—C30	120.1 (3)
P2—Ru2—Ru1	89.629 (19)	C26—C31—H31A	119.9
Ru3—Ru2—Ru1	61.450 (9)	C30—C31—H31A	119.9
C69—Ru3—C68	95.45 (13)	C33—C32—C37	117.5 (3)
C69—Ru3—C70	88.24 (13)	C33—C32—C29	122.5 (3)
C68—Ru3—C70	175.47 (12)	C37—C32—C29	120.0 (3)
C69—Ru3—As1	98.37 (10)	C32—C33—C34	121.4 (4)
C68—Ru3—As1	90.29 (9)	C32—C33—H33A	119.3
C70—Ru3—As1	91.77 (10)	C34—C33—H33A	119.3
C69—Ru3—Ru2	89.80 (10)	C35—C34—C33	120.6 (4)
C68—Ru3—Ru2	96.06 (9)	C35—C34—H34A	119.7
C70—Ru3—Ru2	81.31 (9)	C33—C34—H34A	119.7
As1—Ru3—Ru2	169.149 (12)	C34—C35—C36	118.8 (3)
C69—Ru3—Ru1	147.65 (10)	C34—C35—H35A	120.6
C68—Ru3—Ru1	79.45 (9)	C36—C35—H35A	120.6
C70—Ru3—Ru1	96.03 (8)	C35—C36—C37	120.7 (4)
As1—Ru3—Ru1	113.473 (11)	C35—C36—H36A	119.6
Ru2—Ru3—Ru1	59.452 (8)	C37—C36—H36A	119.6
C38—As1—C50	101.53 (12)	C32—C37—C36	120.9 (4)
C38—As1—C26	100.80 (13)	C32—C37—H37A	119.5
C50—As1—C26	101.54 (12)	C36—C37—H37A	119.5
C38—As1—Ru3	111.63 (9)	C43—C38—C39	118.8 (3)
C50—As1—Ru3	113.18 (9)	C43—C38—As1	120.2 (2)
C26—As1—Ru3	125.09 (9)	C39—C38—As1	120.9 (2)
C1—P1—C7	99.95 (13)	C40—C39—C38	120.4 (3)
C1—P1—C13	102.47 (13)	C40—C39—H39A	119.8
C7—P1—C13	102.02 (14)	C38—C39—H39A	119.8
C1—P1—Ru1	114.45 (10)	C39—C40—C41	121.3 (3)
C7—P1—Ru1	120.11 (10)	C39—C40—H40A	119.3
C13—P1—Ru1	115.25 (9)	C41—C40—H40A	119.3
C20—P2—C14	103.80 (14)	C40—C41—C42	118.0 (3)
C20—P2—C13	105.34 (13)	C40—C41—C44	121.5 (3)
C14—P2—C13	100.07 (12)	C42—C41—C44	120.5 (3)
C20—P2—Ru2	118.69 (10)	C43—C42—C41	120.8 (3)
C14—P2—Ru2	117.18 (10)	C43—C42—H42A	119.6
C13—P2—Ru2	109.61 (10)	C41—C42—H42A	119.6
C2—C1—C6	117.8 (3)	C42—C43—C38	120.6 (3)
C2—C1—P1	125.0 (2)	C42—C43—H43A	119.7
C6—C1—P1	117.2 (2)	C38—C43—H43A	119.7
C1—C2—C3	120.9 (3)	C49—C44—C45	118.5 (3)
C1—C2—H2A	119.5	C49—C44—C41	120.6 (3)
C3—C2—H2A	119.5	C45—C44—C41	121.0 (3)
C4—C3—C2	120.1 (3)	C46—C45—C44	120.7 (3)
C4—C3—H3A	119.9	C46—C45—H45A	119.6
C2—C3—H3A	119.9	C44—C45—H45A	119.6
C3—C4—C5	119.6 (3)	C47—C46—C45	119.7 (3)
C3—C4—H4A	120.2	C47—C46—H46A	120.2
C5—C4—H4A	120.2	C45—C46—H46A	120.2
C4—C5—C6	120.8 (3)	C48—C47—C46	120.1 (3)
C4—C5—H5A	119.6	C48—C47—H47A	119.9
C6—C5—H5A	119.6	C46—C47—H47A	119.9
C5—C6—C1	120.7 (3)	C47—C48—C49	120.5 (4)
C5—C6—H6A	119.7	C47—C48—H48A	119.7
C1—C6—H6A	119.7	C49—C48—H48A	119.7
C8—C7—C12	119.1 (3)	C48—C49—C44	120.4 (3)
C8—C7—P1	119.1 (2)	C48—C49—H49A	119.8
C12—C7—P1	121.8 (2)	C44—C49—H49A	119.8
C9—C8—C7	120.5 (3)	C51—C50—C55	118.2 (3)
C9—C8—H8A	119.8	C51—C50—As1	120.2 (2)
C7—C8—H8A	119.8	C55—C50—As1	121.7 (2)
C10—C9—C8	120.0 (3)	C52—C51—C50	120.9 (3)
C10—C9—H9A	120.0	C52—C51—H51A	119.5
C8—C9—H9A	120.0	C50—C51—H51A	119.5
C9—C10—C11	120.1 (3)	C51—C52—C53	121.7 (3)
C9—C10—H10A	119.9	C51—C52—H52A	119.2
C11—C10—H10A	119.9	C53—C52—H52A	119.2
C12—C11—C10	120.5 (3)	C52—C53—C54	117.4 (3)
C12—C11—H11A	119.7	C52—C53—C56	120.9 (3)
C10—C11—H11A	119.7	C54—C53—C56	121.7 (3)
C11—C12—C7	119.8 (3)	C55—C54—C53	121.0 (3)
C11—C12—H12A	120.1	C55—C54—H54A	119.5
C7—C12—H12A	120.1	C53—C54—H54A	119.5
P2—C13—P1	113.20 (14)	C54—C55—C50	120.7 (3)
P2—C13—H13A	108.9	C54—C55—H55A	119.6
P1—C13—H13A	108.9	C50—C55—H55A	119.6
P2—C13—H13B	108.9	C57—C56—C61	117.6 (3)
P1—C13—H13B	108.9	C57—C56—C53	120.8 (3)
H13A—C13—H13B	107.8	C61—C56—C53	121.6 (3)
C19—C14—C15	119.7 (3)	C56—C57—C58	122.1 (4)
C19—C14—P2	122.4 (2)	C56—C57—H57A	118.9
C15—C14—P2	117.9 (2)	C58—C57—H57A	118.9
C16—C15—C14	119.8 (3)	C59—C58—C57	119.3 (4)
C16—C15—H15A	120.1	C59—C58—H58A	120.4
C14—C15—H15A	120.1	C57—C58—H58A	120.4
C17—C16—C15	120.2 (3)	C58—C59—C60	120.2 (3)
C17—C16—H16A	119.9	C58—C59—H59A	119.9
C15—C16—H16A	119.9	C60—C59—H59A	119.9
C16—C17—C18	120.4 (3)	C59—C60—C61	120.9 (4)
C16—C17—H17A	119.8	C59—C60—H60A	119.5
C18—C17—H17A	119.8	C61—C60—H60A	119.5
C17—C18—C19	119.7 (3)	C60—C61—C56	119.9 (4)
C17—C18—H18A	120.2	C60—C61—H61A	120.1
C19—C18—H18A	120.2	C56—C61—H61A	120.1
C14—C19—C18	120.3 (3)	O1—C62—Ru1	175.3 (3)
C14—C19—H19A	119.8	O2—C63—Ru1	177.1 (3)
C18—C19—H19A	119.8	O3—C64—Ru1	173.2 (2)
C21—C20—C25	118.1 (3)	O4—C65—Ru2	174.2 (2)
C21—C20—P2	123.0 (2)	O5—C66—Ru2	177.4 (3)
C25—C20—P2	118.8 (2)	O6—C67—Ru2	173.4 (3)
C22—C21—C20	121.3 (3)	O7—C68—Ru3	172.4 (3)
C22—C21—H21A	119.4	O8—C69—Ru3	175.8 (3)
C20—C21—H21A	119.4	O9—C70—Ru3	173.6 (3)
			
C63—Ru1—Ru2—C66	−82.9 (6)	C13—P1—C7—C8	−59.8 (3)
C64—Ru1—Ru2—C66	33.1 (3)	Ru1—P1—C7—C8	171.3 (2)
C62—Ru1—Ru2—C66	−138.1 (3)	C1—P1—C7—C12	−137.9 (3)
P1—Ru1—Ru2—C66	125.1 (3)	C13—P1—C7—C12	116.9 (3)
Ru3—Ru1—Ru2—C66	−38.6 (3)	Ru1—P1—C7—C12	−12.0 (3)
C63—Ru1—Ru2—C67	29.0 (5)	C12—C7—C8—C9	−1.4 (5)
C64—Ru1—Ru2—C67	144.98 (13)	P1—C7—C8—C9	175.4 (3)
C62—Ru1—Ru2—C67	−26.22 (13)	C7—C8—C9—C10	0.7 (6)
P1—Ru1—Ru2—C67	−122.98 (10)	C8—C9—C10—C11	0.6 (6)
Ru3—Ru1—Ru2—C67	73.35 (9)	C9—C10—C11—C12	−1.1 (6)
C63—Ru1—Ru2—C65	−144.5 (5)	C10—C11—C12—C7	0.4 (5)
C64—Ru1—Ru2—C65	−28.56 (13)	C8—C7—C12—C11	0.9 (5)
C62—Ru1—Ru2—C65	160.23 (13)	P1—C7—C12—C11	−175.8 (3)
P1—Ru1—Ru2—C65	63.47 (9)	C20—P2—C13—P1	82.02 (18)
Ru3—Ru1—Ru2—C65	−100.20 (9)	C14—P2—C13—P1	−170.52 (16)
C63—Ru1—Ru2—P2	125.3 (5)	Ru2—P2—C13—P1	−46.75 (17)
C64—Ru1—Ru2—P2	−118.72 (9)	C1—P1—C13—P2	145.82 (16)
C62—Ru1—Ru2—P2	70.08 (9)	C7—P1—C13—P2	−111.01 (17)
P1—Ru1—Ru2—P2	−26.68 (3)	Ru1—P1—C13—P2	20.87 (19)
Ru3—Ru1—Ru2—P2	169.65 (2)	C20—P2—C14—C19	13.1 (3)
C63—Ru1—Ru2—Ru3	−44.3 (5)	C13—P2—C14—C19	−95.6 (3)
C64—Ru1—Ru2—Ru3	71.63 (9)	Ru2—P2—C14—C19	146.1 (2)
C62—Ru1—Ru2—Ru3	−99.57 (9)	C20—P2—C14—C15	−168.8 (2)
P1—Ru1—Ru2—Ru3	163.67 (2)	C13—P2—C14—C15	82.6 (2)
C66—Ru2—Ru3—C69	−21.45 (14)	Ru2—P2—C14—C15	−35.8 (2)
C67—Ru2—Ru3—C69	65.41 (14)	C19—C14—C15—C16	−0.3 (4)
C65—Ru2—Ru3—C69	−112.95 (13)	P2—C14—C15—C16	−178.5 (2)
P2—Ru2—Ru3—C69	148.73 (11)	C14—C15—C16—C17	−0.5 (4)
Ru1—Ru2—Ru3—C69	169.41 (10)	C15—C16—C17—C18	1.1 (4)
C66—Ru2—Ru3—C68	−116.92 (13)	C16—C17—C18—C19	−0.8 (5)
C67—Ru2—Ru3—C68	−30.05 (13)	C15—C14—C19—C18	0.6 (4)
C65—Ru2—Ru3—C68	151.58 (12)	P2—C14—C19—C18	178.6 (2)
P2—Ru2—Ru3—C68	53.27 (10)	C17—C18—C19—C14	0.0 (5)
Ru1—Ru2—Ru3—C68	73.95 (9)	C14—P2—C20—C21	−102.9 (3)
C66—Ru2—Ru3—C70	66.79 (13)	C13—P2—C20—C21	1.8 (3)
C67—Ru2—Ru3—C70	153.66 (12)	Ru2—P2—C20—C21	124.9 (2)
C65—Ru2—Ru3—C70	−24.71 (12)	C14—P2—C20—C25	77.4 (3)
P2—Ru2—Ru3—C70	−123.02 (9)	C13—P2—C20—C25	−177.9 (2)
Ru1—Ru2—Ru3—C70	−102.34 (8)	Ru2—P2—C20—C25	−54.7 (3)
C66—Ru2—Ru3—As1	117.60 (12)	C25—C20—C21—C22	1.3 (5)
C67—Ru2—Ru3—As1	−155.54 (11)	P2—C20—C21—C22	−178.4 (2)
C65—Ru2—Ru3—As1	26.10 (10)	C20—C21—C22—C23	−0.6 (5)
P2—Ru2—Ru3—As1	−72.22 (8)	C21—C22—C23—C24	−0.2 (5)
Ru1—Ru2—Ru3—As1	−51.54 (6)	C22—C23—C24—C25	0.2 (5)
C66—Ru2—Ru3—Ru1	169.13 (10)	C23—C24—C25—C20	0.5 (5)
C67—Ru2—Ru3—Ru1	−104.00 (9)	C21—C20—C25—C24	−1.2 (5)
C65—Ru2—Ru3—Ru1	77.63 (8)	P2—C20—C25—C24	178.4 (2)
P2—Ru2—Ru3—Ru1	−20.68 (4)	C38—As1—C26—C31	167.7 (2)
C63—Ru1—Ru3—C69	151.5 (2)	C50—As1—C26—C31	−88.0 (3)
C64—Ru1—Ru3—C69	−122.5 (2)	Ru3—As1—C26—C31	41.4 (3)
C62—Ru1—Ru3—C69	57.8 (2)	C38—As1—C26—C27	−11.8 (3)
P1—Ru1—Ru3—C69	−51.18 (19)	C50—As1—C26—C27	92.4 (3)
Ru2—Ru1—Ru3—C69	−20.08 (19)	Ru3—As1—C26—C27	−138.2 (2)
C63—Ru1—Ru3—C68	68.06 (14)	C31—C26—C27—C28	−0.6 (5)
C64—Ru1—Ru3—C68	153.99 (13)	As1—C26—C27—C28	179.0 (2)
C62—Ru1—Ru3—C68	−25.66 (13)	C26—C27—C28—C29	−0.6 (5)
P1—Ru1—Ru3—C68	−134.66 (10)	C27—C28—C29—C30	1.4 (5)
Ru2—Ru1—Ru3—C68	−103.57 (9)	C27—C28—C29—C32	−177.3 (3)
C63—Ru1—Ru3—C70	−112.19 (14)	C28—C29—C30—C31	−1.0 (5)
C64—Ru1—Ru3—C70	−26.27 (13)	C32—C29—C30—C31	177.7 (3)
C62—Ru1—Ru3—C70	154.09 (13)	C27—C26—C31—C30	1.0 (5)
P1—Ru1—Ru3—C70	45.08 (10)	As1—C26—C31—C30	−178.6 (2)
Ru2—Ru1—Ru3—C70	76.18 (9)	C29—C30—C31—C26	−0.2 (5)
C63—Ru1—Ru3—As1	−17.62 (10)	C30—C29—C32—C33	130.4 (4)
C64—Ru1—Ru3—As1	68.30 (9)	C28—C29—C32—C33	−50.9 (5)
C62—Ru1—Ru3—As1	−111.34 (9)	C30—C29—C32—C37	−50.5 (5)
P1—Ru1—Ru3—As1	139.66 (4)	C28—C29—C32—C37	128.2 (4)
Ru2—Ru1—Ru3—As1	170.752 (14)	C37—C32—C33—C34	1.0 (6)
C63—Ru1—Ru3—Ru2	171.63 (10)	C29—C32—C33—C34	−179.9 (4)
C64—Ru1—Ru3—Ru2	−102.45 (9)	C32—C33—C34—C35	−0.3 (7)
C62—Ru1—Ru3—Ru2	77.91 (9)	C33—C34—C35—C36	−1.8 (7)
P1—Ru1—Ru3—Ru2	−31.10 (4)	C34—C35—C36—C37	3.2 (7)
C69—Ru3—As1—C38	65.82 (13)	C33—C32—C37—C36	0.4 (6)
C68—Ru3—As1—C38	161.38 (12)	C29—C32—C37—C36	−178.7 (4)
C70—Ru3—As1—C38	−22.65 (12)	C35—C36—C37—C32	−2.6 (7)
Ru2—Ru3—As1—C38	−72.69 (11)	C50—As1—C38—C43	−165.2 (2)
Ru1—Ru3—As1—C38	−120.01 (9)	C26—As1—C38—C43	−60.9 (3)
C69—Ru3—As1—C50	−47.98 (14)	Ru3—As1—C38—C43	74.0 (2)
C68—Ru3—As1—C50	47.57 (13)	C50—As1—C38—C39	19.8 (3)
C70—Ru3—As1—C50	−136.46 (12)	C26—As1—C38—C39	124.1 (3)
Ru2—Ru3—As1—C50	173.50 (11)	Ru3—As1—C38—C39	−101.0 (2)
Ru1—Ru3—As1—C50	126.18 (9)	C43—C38—C39—C40	−1.3 (5)
C69—Ru3—As1—C26	−172.52 (15)	As1—C38—C39—C40	173.8 (2)
C68—Ru3—As1—C26	−76.96 (14)	C38—C39—C40—C41	1.4 (5)
C70—Ru3—As1—C26	99.00 (14)	C39—C40—C41—C42	0.2 (5)
Ru2—Ru3—As1—C26	48.97 (13)	C39—C40—C41—C44	179.9 (3)
Ru1—Ru3—As1—C26	1.65 (11)	C40—C41—C42—C43	−1.9 (5)
C63—Ru1—P1—C1	75.39 (14)	C44—C41—C42—C43	178.4 (3)
C64—Ru1—P1—C1	−16.59 (13)	C41—C42—C43—C38	2.0 (5)
C62—Ru1—P1—C1	168.35 (13)	C39—C38—C43—C42	−0.4 (5)
Ru2—Ru1—P1—C1	−109.84 (10)	As1—C38—C43—C42	−175.5 (2)
Ru3—Ru1—P1—C1	−83.52 (11)	C40—C41—C44—C49	−137.7 (3)
C63—Ru1—P1—C7	−43.49 (15)	C42—C41—C44—C49	42.1 (4)
C64—Ru1—P1—C7	−135.47 (14)	C40—C41—C44—C45	42.8 (4)
C62—Ru1—P1—C7	49.47 (14)	C42—C41—C44—C45	−137.5 (3)
Ru2—Ru1—P1—C7	131.28 (11)	C49—C44—C45—C46	−0.7 (5)
Ru3—Ru1—P1—C7	157.60 (11)	C41—C44—C45—C46	178.9 (3)
C63—Ru1—P1—C13	−166.15 (15)	C44—C45—C46—C47	0.0 (6)
C64—Ru1—P1—C13	101.87 (13)	C45—C46—C47—C48	1.1 (6)
C62—Ru1—P1—C13	−73.19 (14)	C46—C47—C48—C49	−1.5 (6)
Ru2—Ru1—P1—C13	8.63 (11)	C47—C48—C49—C44	0.8 (6)
Ru3—Ru1—P1—C13	34.95 (12)	C45—C44—C49—C48	0.3 (5)
C66—Ru2—P2—C20	110.86 (14)	C41—C44—C49—C48	−179.2 (3)
C67—Ru2—P2—C20	19.24 (14)	C38—As1—C50—C51	−105.5 (3)
C65—Ru2—P2—C20	−158.89 (13)	C26—As1—C50—C51	150.8 (3)
Ru3—Ru2—P2—C20	−59.31 (12)	Ru3—As1—C50—C51	14.3 (3)
Ru1—Ru2—P2—C20	−77.38 (11)	C38—As1—C50—C55	73.7 (3)
C66—Ru2—P2—C14	−15.08 (15)	C26—As1—C50—C55	−30.0 (3)
C67—Ru2—P2—C14	−106.69 (14)	Ru3—As1—C50—C55	−166.6 (2)
C65—Ru2—P2—C14	75.18 (13)	C55—C50—C51—C52	2.3 (5)
Ru3—Ru2—P2—C14	174.76 (10)	As1—C50—C51—C52	−178.5 (3)
Ru1—Ru2—P2—C14	156.68 (11)	C50—C51—C52—C53	0.8 (5)
C66—Ru2—P2—C13	−128.13 (14)	C51—C52—C53—C54	−3.7 (5)
C67—Ru2—P2—C13	140.25 (13)	C51—C52—C53—C56	174.6 (3)
C65—Ru2—P2—C13	−37.88 (12)	C52—C53—C54—C55	3.4 (5)
Ru3—Ru2—P2—C13	61.70 (11)	C56—C53—C54—C55	−174.9 (3)
Ru1—Ru2—P2—C13	43.63 (10)	C53—C54—C55—C50	−0.4 (5)
C7—P1—C1—C2	−110.8 (3)	C51—C50—C55—C54	−2.6 (5)
C13—P1—C1—C2	−6.0 (3)	As1—C50—C55—C54	178.3 (3)
Ru1—P1—C1—C2	119.5 (3)	C52—C53—C56—C57	−25.7 (5)
C7—P1—C1—C6	70.9 (3)	C54—C53—C56—C57	152.6 (3)
C13—P1—C1—C6	175.7 (3)	C52—C53—C56—C61	153.6 (3)
Ru1—P1—C1—C6	−58.8 (3)	C54—C53—C56—C61	−28.2 (5)
C6—C1—C2—C3	−1.8 (5)	C61—C56—C57—C58	0.3 (5)
P1—C1—C2—C3	179.9 (3)	C53—C56—C57—C58	179.6 (3)
C1—C2—C3—C4	−0.7 (6)	C56—C57—C58—C59	0.8 (5)
C2—C3—C4—C5	2.7 (6)	C57—C58—C59—C60	−1.1 (5)
C3—C4—C5—C6	−2.1 (6)	C58—C59—C60—C61	0.3 (5)
C4—C5—C6—C1	−0.4 (6)	C59—C60—C61—C56	0.8 (5)
C2—C1—C6—C5	2.4 (5)	C57—C56—C61—C60	−1.1 (5)
P1—C1—C6—C5	−179.2 (3)	C53—C56—C61—C60	179.6 (3)
C1—P1—C7—C8	45.4 (3)		

### Hydrogen-bond geometry (Å, °)

**Table:**

Cg1 and Cg2 are the centroids of the C7–C12 and C26–C31 benzene rings, respectively.

**Table:**

*D*—H···*A*	*D*—H	H···*A*	*D*···*A*	*D*—H···*A*
C46—H46A···O9^i^	0.93	2.56	3.249 (4)	131
C21—H21A···Cg1	0.93	2.95	3.707 (4)	139
C24—H24A···Cg2^ii^	0.93	2.92	3.582 (4)	129

Symmetry codes: (i) −*x*, −*y*+1, −*z*;  (ii) *x*+1, *y*, *z*.
